# Quantitative muscle MRI combined with AI‐based segmentation as a follow‐up biomarker for ATTRv patients: A longitudinal pilot study

**DOI:** 10.1111/ene.16574

**Published:** 2024-11-27

**Authors:** Etienne Fortanier, Constance P. Michel, Marc Adrien Hostin, Emilien Delmont, Annie Verschueren, Maxime Guye, Marc‐Emmanuel Bellemare, David Bendahan, Shahram Attarian

**Affiliations:** ^1^ Reference Center for Neuromuscular Diseases and ALS La Timone University Hospital, Aix‐Marseille University Marseille France; ^2^ Center for Magnetic Resonance in Biology and Medicine Aix‐Marseille University, UMR CNRS 7339 Marseille France; ^3^ Aix Marseille University, CNRS, LIS Marseille France; ^4^ Medicine Faculty Aix‐Marseille University, UMR 7286 Marseille France; ^5^ Aix‐Marseille University, Inserm, GMGF Marseille France

**Keywords:** amyloid, automated, biomarkers, fat fraction, imaging, longitudinal, MRI, neuropathy, quantitative

## Abstract

**Background and Objectives:**

Intramuscular fat fraction (FF), assessed using quantitative MRI (qMRI), has emerged as a promising biomarker for hereditary transthyretin amyloidosis (ATTRv) patients. Currently, the main drawbacks to its use in future therapeutic trials are its sensitivity to change over a short period of time and the time‐consuming manual segmentation step to extract quantitative data. This pilot study aimed to demonstrate the suitability of an Artificial Intelligence‐based (AI) segmentation technique to assess disease progression in a real‐life cohort of ATTRv patients over 1 year.

**Methods:**

Fifteen ATTRv patients were included in this monocentric, observational, prospective study. FF, magnetization transfer ratio (MTR), and quantitative T2 were extracted from patients' lower limb qMRI at two time points, 1 year apart, at thigh and leg levels. qMRI parameters were correlated with clinical and electrophysiological parameters assessed at the same time.

**Results:**

Global FF at leg level significantly progressed over 1 year: +1.28 ± 2.62% (*p* = 0.017). At thigh level, no significant change in global FF, MTR, or T2 was measured. The leg FF was strongly correlated with the main clinical and electrophysiological scores.

**Conclusion:**

AI‐based CNN network segmentation combined with qMRI can be used to obtain quantitative metrics for longitudinal studies in ATTRv patients. Global FF at the leg level seems to be the most sensitive MRI biomarker to track disease progression in a 1‐year period. Larger studies with treatment‐specific groups will now be necessary to determine the place of qMRI markers compared to the current clinical and electrophysiological scores.

## INTRODUCTION

Hereditary transthyretin amyloidosis (ATTRv) is an inherited disease caused by mutations in the transthyretin (TTR) gene [1]. These neuropathies are severe and disabling but a number of therapies are now available, including TTR stabilizers and RNA interference (RNAi)‐based drugs that can slow the progression of the disease [2]. In order to determine the efficiency of these new therapeutic strategies, highly sensitive clinical and paraclinical tools are needed to assess the corresponding changes over short periods of time. Neurological damage in ATTRv patients is currently monitored through clinical, biological, and electrophysiological investigations. However, the corresponding sensitivity is shown to be limited in this disease [3].

In recent years, quantitative neuromuscular imaging techniques have been developed with the aim of assessing in vivo structural changes and providing quantitative biomarkers for muscle and nerve tissues [4, 5]. In ATTRv patients, quantitative muscle MRI (qMRI) in the lower limbs has shown significant differences in several metrics such as intramuscular fat fraction (FF) and muscle magnetization transfer ratio (MTR) between controls, asymptomatic carriers, and symptomatic patients [6, 7]. The precise role of qMRI in the management of ATTRv patients remains to be confirmed through dedicated longitudinal multimodal studies comparing the different clinical, electrophysiological, and biological biomarkers.

From a technical point of view, the bottleneck of qMRI is the segmentation step needed to delineate the different regions of interest (ROI) [8, 9]. Since a manual process is prone to error and is very time‐consuming, several automatic AI‐based segmentation tools, such as convolutional neural networks (CNN), have been developed [10] and applied in neuromuscular patients [11]. In particular, the nnU‐Net network offers an easy‐to‐use, automatically parameterized framework with excellent segmentation results in several cohorts, bringing this technique closer to use in clinical practice [12].

In the present pilot study, we intended to assess the suitability of a fully automated AI‐based segmentation technique combined with qMRI in order to evaluate longitudinal changes and provide sensitive biomarkers in the lower limb individual muscles of ATTRv patients over a 1‐year period.

## PATIENTS AND METHODS

### Study design and participants

We conducted a single‐center, prospective, observational, pilot study. ATTRv patients with genetically confirmed TTR mutation were recruited in the Reference Center for Neuromuscular Diseases (Marseille‐France) between May 2017 and December 2021 (IRB #2015‐A00799‐40). No participants had any history of other neuromuscular disorders and other conditions responsible for peripheral neuropathy were excluded.

Clinical severity was assessed twice, that is, at baseline (P0) and follow‐up visits (P1) using the Polyneuropathy Disability (PND), the Neuropathy Impairment Score (NIS/244) with the lower limb subscore (NIS‐LL/88), the Overall Neuropathy Limitation Scale (ONLS), and the Rasch‐built Overall Disability Scale (R‐ODS/48). The Medical Research Council (MRC) scale was used to assess muscle strength in the lower limb on 4 muscle groups: psoas, quadriceps, tibialis anterior (TA), and plantar flexor (corresponding maximum score/20).

Electrodiagnosis (EDX) was performed on the tibial and fibular motor nerves and the sural nerve of the non‐dominant lower limb (Medtronic Keypoint G4 device). Distal motor amplitudes (CMAP) and sural sensory nerve action potential (SNAP) were recorded. Motor units were counted using the MUNIX (motor unit index) method on the TA muscle (MUNIX TA) [13]. Due to COVID restrictions, the follow‐up interval was delayed for a few patients and the whole set of clinical and MRI data was standardized to a 12‐month interval.

### MRI and AI‐based segmentation protocol

**FIGURE 1:**
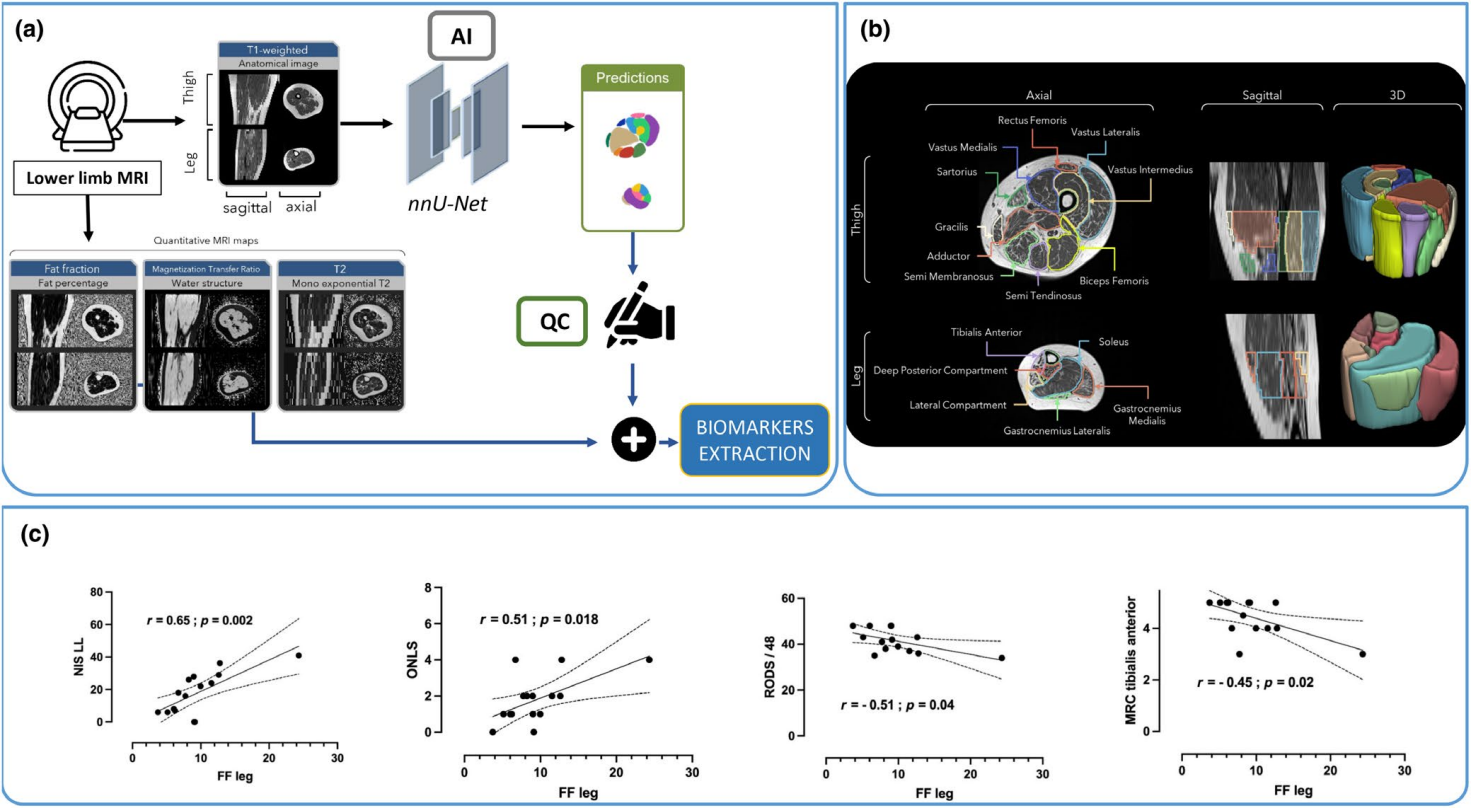
(a) Schematic representation of the imaging pipeline used to extract quantitative MRI data. Individual muscles were segmented using automatic AI‐based predictions with nnU‐Net (with a manual quality check). Using image registration methods, masks obtained were then projected onto the quantitative MRI maps so as to quantify FF, MTR, and T2‐W in individual muscles of both thigh and leg. (b) Example of T1W axial (left) and sagittal (middle) images from an ATTRv patient recorded at thigh (upper panels) and leg (lower panels) levels at baseline. Eleven masks are identified in the thigh and six in the leg with dedicated colors. Schematic 3D representation of all the masks is shown in the right panels. (c) Correlation between Fat Fraction at leg level and clinical parameters. Rho and *p*‐values are indicated for each correlation. FF, fat fraction; MRC, Medical Research Council; NIS‐LL, Neuropathy Impairment Score lower limb subscore; ONLS, Overall Neuropathy limitation scale; RODS, Rasch‐built overall disability scale.

ATTRv patients were positioned supine for magnetic resonance imaging of the leg and thigh of the non‐dominant limb using a flexible radiofrequency coil array. Image sets were acquired in the axial plane at 1.5 T (MAGNETOM Avanto, Siemens Healthineers, Erlangen, Germany) during approximately 45 min. Anatomical (T1) and quantitative imaging sequences (including three‐point‐Dixon) were used to compute three metrics from the generation of quantitative maps: FF, quantitative water T2 (T2‐W), and magnetization transfer ratio (MTR) as previously described [6]. Ten thigh and six leg muscle regions were segmented using a nnU‐Net network (Figure 1a,b) trained with parameters obtained from a 218 MRI acquisitions dataset analyzed previously [14]. These AI‐based segmentations were quality checked (QC) for all subjects by the same blinded operator (EF). Time efficiency and segmentation performances of this network are detailed in another cohort from our center [12]. Segmented masks were then resampled and registered to the quantitative maps and the mean values within each ROI and each slice were computed. The same protocol was performed at baseline and follow‐up evaluations.

### Statistical analyses

Depending on the distribution, quantitative data were expressed as medians (Interquartile) or mean (SD). Comparative analyses were performed either using nonparametric Wilcoxon tests or Student *t*‐test for paired data. Spearman's correlation coefficients or linear regression were used to compare imaging and clinical biomarkers at baseline. Differences were considered statistically significant for *p*‐values below 0.05. Statistical analyses were performed using RStudio.

## RESULTS

### Demographic, clinical, and electrophysiological data

Fifteen ATTRv patients (12 males) with confirmed genetic diagnosis were included in this study. Median age at enrollment was 49 (38–58.5) years and median disease duration was 2 (1–4) years. TTR mutations included: *Val30Met* (10 patients), *Glu89Gln* (2), *Phe64Leu* (1), *Ser77Phe* (1), and *Val107Ile* (1). Patients were treated with TTR stabilizers (8), liver transplantation (2), or RNAi drugs (6). Median clinical scores at baseline were: PND score: 1 (1–2), NIS: 31 (12–40), NIS‐LL: 18 (7–26), ONLS: 2 (1–2), and RODS: 42 (37–48). Total MRC score (/20) in the lower limb was 19 (18–20), and 5 (4–5), in the TA muscle (/5).

Baseline EDX results were: fibular CMAP 1 mV (0.2–2.8), tibial CMAP 0.35 mV (0.1–5.5), CMAP sum score 1.3 mV (0.5–7.5), sural SNAP 4.9 μV (0.‐11), and MUNIX TA 84 (61–102).

None of the clinical scores significantly changed over the 1‐year period: PND (*p* = 0.317), NIS (*p* = 0.875), NIS‐LL (*p* = 0.082), ONLS (*p* = 0.891), RODS (*p* = 0.574), total MRC (*p* = 0.675) and TA MRC (*p* = 0.317). Electrophysiological parameters at P1 showed a significant decrease for tibial CMAP −0.3 mV (−0.3.−0.8, *p* = 0.005), CMAP sum score − 0.5 mV (−0.1.−1.3, *p* = 0.010) and sural SNAP −0.9 μV (−0.0.−4.9, *p* = 0.008).

### Longitudinal MRI biomarkers progression

At baseline, the average FF at leg and thigh levels were 9.48 ± 4.88% and 8.03 ± 3.86%, respectively (Table 1).

**TABLE 1:** Longitudinal changes of MRI biomarkers over a 1‐year period in ATTRv‐PN patients.

Section	Visit	P0			P1			P1‐P0		
	Metric	FF	MTR	T2‐W	FF	MTR	T2‐W	FF	MTR	T2‐W
	Label									
Leg	Xh	9.48 ± 4.88	45.58 ± 4.10	31.26 ± 2.61	10.76 ± 6.69	45.00 ± 5.33	30.38 ± 2.12	1.28 ± 2.62*	−0.58 ± 1.91	−0.88 ± 1.73
	DPC	8.07 ± 3.42	44.59 ± 3.55	31.07 ± 2.08	8.87 ± 4.29	44.61 ± 4.42	30.61 ± 1.34	0.80 ± 1.65*	0.02 ± 1.91	−0.46 ± 1.34
	GL	11.31 ± 6.58	43.70 ± 5.72	29.83 ± 3.43	12.51 ± 8.09	43.33 ± 6.56	29.25 ± 3.52	1.20 ± 3.03	−0.37 ± 2.26	−0.58 ± 3.51
	GM	9.36 ± 4.48	44.09 ± 4.85	31.72 ± 3.56	11.06 ± 6.37	43.22 ± 4.93	30.92 ± 3.40	1.69 ± 3.00*	−0.87 ± 2.01	−0.81 ± 2.27
	LC	12.98 ± 7.21	44.83 ± 6.63	29.26 ± 2.64	14.50 ± 10.73	43.84 ± 8.96	28.73 ± 2.38	1.52 ± 4.10	−0.99 ± 3.15	−0.52 ± 2.06
	So	9.54 ± 6.09	45.99 ± 4.11	32.05 ± 2.87	10.82 ± 7.72	45.48 ± 5.55	30.97 ± 2.45	1.29 ± 2.82*	−0.51 ± 2.19	−1.08 ± 1.88*
	TA	8.49 ± 3.58	46.81 ± 3.82	31.02 ± 4.19	9.74 ± 5.26	46.10 ± 5.05	29.91 ± 2.96	1.25 ± 2.47*	−0.72 ± 2.09	−1.11 ± 2.02
Thigh	All	8.03 ± 3.86	47.48 ± 2.59	29.32 ± 2.33	8.32 ± 4.12	47.58 ± 2.68	29.55 ± 2.24	0.29 ± 0.76	0.10 ± 1.10	0.23 ± 1.61
	Ad	8.95 ± 4.83	47.50 ± 2.96	29.72 ± 2.48	9.24 ± 4.81	47.33 ± 3.03	29.91 ± 2.60	0.29 ± 0.74	−0.17 ± 0.86	0.19 ± 1.90
	BF	8.75 ± 5.00	46.60 ± 3.58	28.71 ± 2.96	9.07 ± 6.01	46.78 ± 3.56	29.25 ± 3.23	0.32 ± 1.62	0.18 ± 1.21	0.54 ± 1.69
	Gr	8.16 ± 5.02	47.65 ± 3.75	23.83 ± 2.33	8.59 ± 5.81	47.23 ± 3.99	24.44 ± 2.40	0.44 ± 1.09	−0.43 ± 1.44	0.62 ± 1.17
	RF	5.38 ± 2.48	47.10 ± 2.42	27.42 ± 4.16	5.48 ± 2.64	46.93 ± 2.61	26.76 ± 3.30	0.10 ± 0.52	−0.17 ± 2.56	−0.66 ± 3.39
	SM	9.87 ± 8.05	46.99 ± 5.47	28.40 ± 2.61	10.19 ± 8.66	46.92 ± 5.84	29.22 ± 2.84	0.32 ± 1.21	−0.07 ± 1.13	0.82 ± 0.88*
	ST	8.64 ± 5.74	47.25 ± 3.45	26.86 ± 2.42	8.81 ± 6.15	47.17 ± 3.88	27.66 ± 2.53	0.17 ± 1.54	−0.08 ± 1.35	0.80 ± 1.60
	Sa	10.94 ± 5.74	45.47 ± 2.94	26.39 ± 2.32	11.24 ± 6.23	45.66 ± 4.26	26.60 ± 2.17	0.30 ± 1.81	0.19 ± 1.83	0.22 ± 2.06
	VI	7.01 ± 3.87	48.50 ± 2.95	30.80 ± 2.53	7.45 ± 4.13	48.69 ± 3.08	30.99 ± 2.47	0.44 ± 0.76*	0.19 ± 1.18	0.19 ± 1.48
	VL	8.39 ± 3.71	46.68 ± 3.32	30.41 ± 2.89	8.78 ± 3.82	46.94 ± 3.38	30.27 ± 2.49	0.40 ± 0.77	0.26 ± 1.24	−0.15 ± 2.01
	VM	5.69 ± 2.18	48.92 ± 1.80	30.84 ± 3.10	5.80 ± 2.26	49.29 ± 1.98	30.97 ± 2.70	0.10 ± 0.56	0.37 ± 1.61	0.14 ± 2.09

*Note:* Results are shown as mean ± SD. *refers to significant differences between P0 and P1 values.

Abbreviations: Ad: Adductor, All: Whole‐Leg, All: whole set of muscles for Leg or thigh, BF: Biceps Femoris, DPC: Deep Posterior Compartment, FF: Fat Fraction, GL: Gastrocnemius Lateralis, GM: Gastrocnemius Medialis, Gr: Gracilis, LC: Lateral Compartment, MTR: Magnetization Transfert Ratio, RF: Rectus Femoris, Sa: Sartorius, SM: Semi Membranosus, So: Soleus, ST: Semi Tendinosus, TA: Tibialis anterior, VI: Vastus Intermedius, VL: Vastus Lateralis, VM: Vastus Medialis.

Whole‐leg FF change was significant over the 1‐year period with a + 1.28 ± 2.62% increase (*p* = 0.034). This FF progression was significant in the Deep posterior compartment (+0.80 ± 1.65%, *p* = 0.012), the Gastrocnemius Medialis (+1.69 ± 3.00%, *p* = 0.007), the Soleus (+1.29 ± 2.82%, *p* = 0.007) and the TA (+1.25 ± 2.47%, *p* = 0.018). At the thigh level, a significant FF increase was only measured in the Vastus Intermedius (+0.44 ± 0.76%, *p* = 0.009). A significant T2‐W decrease was observed in the Soleus (−1.08 ± 1.88%, *p* = 0.008) while an increase was measured in the Semi Tendinosus (+0.82 ± 0.88%, *p* = 0.009). No significant change was observed for the MTR in any muscle.

### Correlation between MRI biomarkers and clinical parameters

Whole‐leg FF was correlated with the main clinical features: NIS‐LL (rho = 0.65; *p* = 0.002), ONLS scale (rho = 0.51; *p* = 0.018), R‐ODS (rho = −0.41; *p* = 0.040) and MRC in the TA muscle (rho = −0.45; *p* = 0.020) (Figure 1c). Global leg FF was also correlated with the MUNIX in the TA (rho = −0.64; *p* = 0.010).

## DISCUSSION

In this pilot study, we investigated the suitability of qMRI metrics for assessing disease progression in ATTRv patients over a 1‐year period. These metrics were extracted using a newly designed quantitative imaging pipeline including nnU‐Net to perform a fully automatic segmentation. The main finding was a significant FF progression at leg level (+1.28%/year) while no significant FF change was measured at thigh level. No major changes were observed in MTR and T2‐W. Interestingly, none of the clinical scores changed over this short period. On the contrary, a few EDX parameters (tibial CMAP, CMAP sum score, and sural SNAP) showed a significant slight deterioration over 1 year.

The interest in imaging parameters in ATTRv patients has been reported in a few cross‐sectional studies but has never been documented in longitudinal studies [6, 7]. The present study identified FF at the leg level as the most interesting metric, with significant correlations with the main clinical and electrophysiological parameters. These findings further support those previously reported for the longitudinal assessment of hereditary neuropathies in CMT1A patients using qMRI combined with an automated segmentation process [11, 12]. In addition to the drastic gain of time for the segmentation process (9 times quicker in our previous study on CMT1A patients [12]), the AI‐based segmentation method offers a very robust analytical frame by avoiding the bias related to manual segmentation [15].

This work was a pilot study with several limitations: it was a single‐center with a small sample size, and heterogeneity among patients regarding their treatment and their genetic phenotype. The next step will be the application of this method to a multicentric study with dedicated treatment‐specific groups of patients.

In conclusion, this AI‐based segmentation technique applied to neuromuscular qMRI is ready to be used in longitudinal studies to assess disease severity and progression in ATTRv patients. The exact place for this tool for monitoring ATTRv patients remains to be demonstrated, but it is now possible to consider its use in future clinical studies.

## AUTHOR CONTRIBUTIONS

**Etienne Fortanier:** Conceptualization; investigation; funding acquisition; writing – original draft; data curation; methodology. **Constance P. Michel:** Investigation; methodology; formal analysis; software; data curation. **Marc Adrien Hostin:** Data curation; software; formal analysis; methodology; investigation. **Emilien Delmont:** Writing – original draft; data curation; validation; methodology. **Annie Verschueren:** Conceptualization; investigation. **Maxime Guye:** Conceptualization; investigation. **Marc‐Emmanuel Bellemare:** Conceptualization; supervision; writing – review and editing. **David Bendahan:** Writing – review and editing; supervision; data curation; software; writing – original draft. **Shahram Attarian:** Validation; methodology; writing – review and editing; data curation; supervision.

## CONFLICT OF INTEREST STATEMENT

All authors have no conflicts of interest.

## Data Availability

The data that support the findings of this study are available from the corresponding author upon reasonable request.
